# Breast cancer with extensive bone metastasis mimicking myeloma

**DOI:** 10.1002/ccr3.817

**Published:** 2017-01-17

**Authors:** Ya‐Ting Hsu, Kung‐Chao Chang

**Affiliations:** ^1^ Departments of Internal MedicineCollege of MedicineNational Cheng Kung University HospitalNational Cheng Kung UniversityTainanTaiwan; ^2^ PathologyCollege of MedicineNational Cheng Kung University HospitalNational Cheng Kung UniversityTainanTaiwan

**Keywords:** Breast cancer, metastasis, myeloma, punched‐out, skull

## Abstract

Punched‐out lesions in skull film usually elicit consideration of myeloma. However, some other diseases cause similar clinical presentations. We describe an uncommon presentation of breast cancer, which mimics multiple myeloma. Therefore, in a broad spectrum of clinical settings, physicians should always consider differential diagnosis and await for definitive pathological diagnosis.

## Question

**Figure 1:**
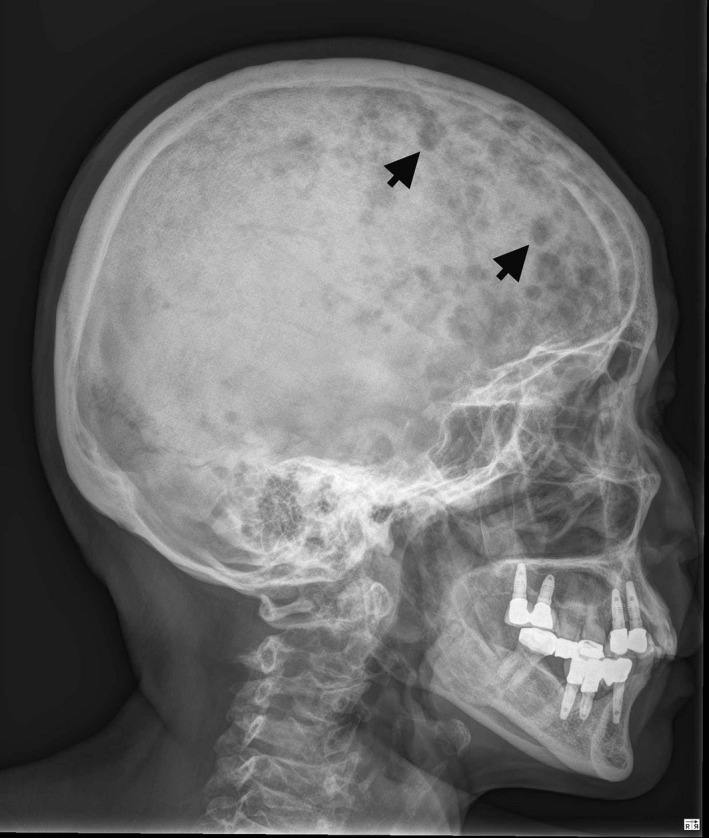
X‐ray of skull bone shows multiple punched‐out lesions (arrows).

A 52‐years‐old woman presented with progressive bone pain for 3 months. A skull film is shown in Fig. 1. Laboratory data exhibited normocytic anemia and elevated serum *β*2‐microglobulin. What is your diagnosis?

## Diagnosis

**Figure 2:**
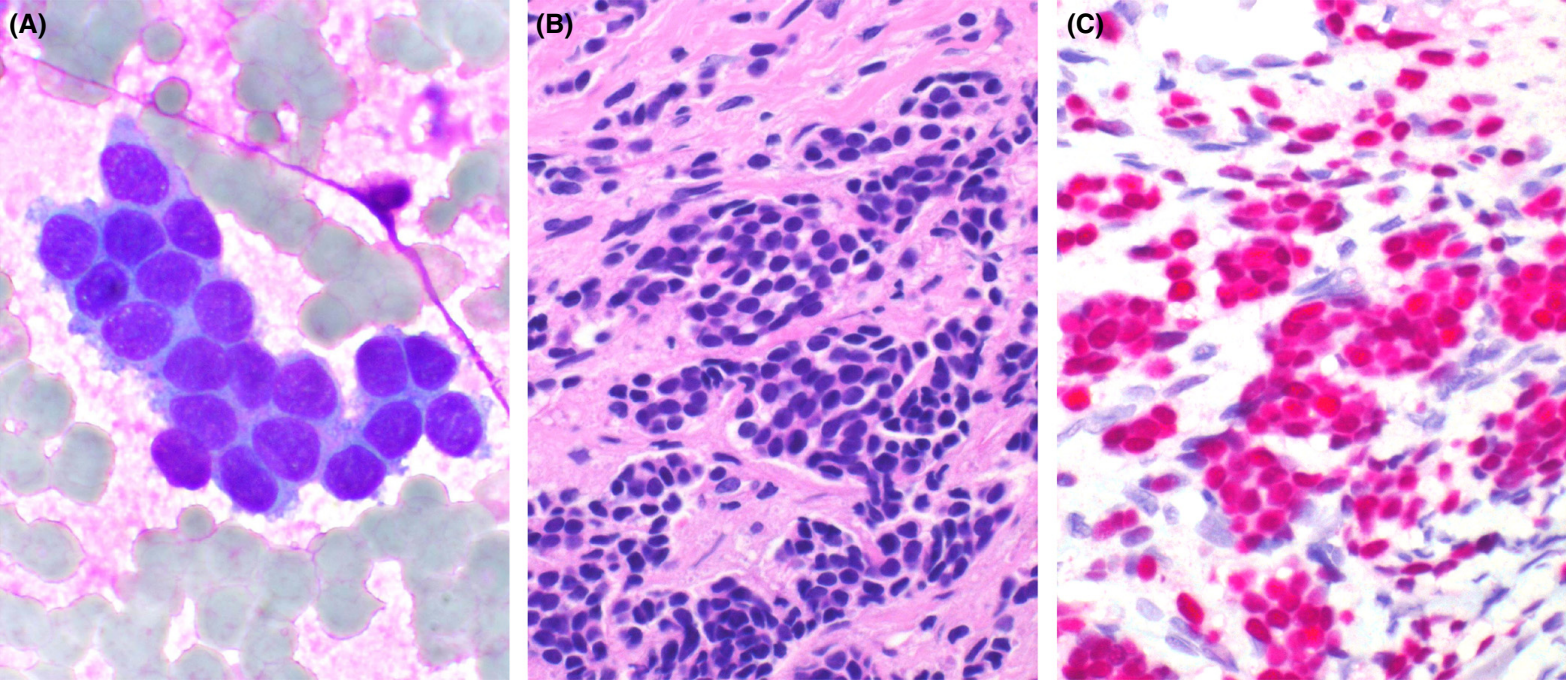
(A) Bone marrow smear shows tumor cells arranged in solid nests with nuclear hyperchromatism (Liu stain, 1000×). (B) Bone marrow biopsy reveals cell nests of metastatic carcinoma (H&E stain, 400×). (C) Immunohistochemically, the tumor cells express estrogen receptor (red color, hematoxylin counterstain, 400×).

Considering multiple myeloma, bone marrow examination was carried out and showed tumor cells arranged in solid nests (Fig. 2). Immunofixation electrophoresis was negative. Bone marrow biopsy showed metastatic carcinoma positive for estrogen receptor (Fig. 2) and progesterone receptor, consistent with invasive lobular carcinoma of breast. Chest CT revealed an ill‐defined tumor (4.4 cm) occupying the outer aspect of right breast with multicentric appearance. Staging workup revealed bone metastasis without solid organ involvement. Currently, the patient is undergoing chemotherapy, hormone therapy, and bisphosphonate with relief of back pain.

## Authorship

Hsu YT: wrote the article. Chang KC: supervised and wrote the article.

## Conflict of Interest

None declared.

